# The Persian Checklist of Pleasant Events (PCPE): Development, Validity and Reliability

**Published:** 2015-09

**Authors:** Sepideh Bakht, Tahereh Mahdavi Haji, Ensiyeh Ghasemian Shirvan, Hamed Ekhtiari

**Affiliations:** 1 Translational Neuroscience Program, Institute for Cognitive Sciences Studies (ICSS), Tehran, Iran; 2 Neurocognitive Laboratory, Iranian National Center for Addiction Studies, Tehran University of Medical Sciences, Tehran, Iran

**Keywords:** *Behavioral Activation*, *Iran*, *Pleasant Events*, *Pleasant Events Schedule*, *Reliability*, *Validity*

## Abstract

**Objective:** Experiencing ‎pleasant events during daily life ‎has a significant positive role in ‎the personal mental health and ‎acts as a keystone for “behavioral ‎activation” (BA) interventions. ‎There are serious differences in ‎the pleasant event schedules in ‎different cultures and countries. ‎We aimed to develop a Persian ‎checklist of pleasant events ‎‎(PCPE) to provide and validate a ‎culturally compatible checklist for ‎Iranians.‎

**Methods:** To develop a checklist ‎of pleasant events, inspired by ‎Pleasant Events Schedule (PES) ‎‎(MacPhillamy & Lewinsohn, ‎‎1982), we held three focused ‎group discussions with 24 normal ‎healthy participants from both ‎genders (female = 12) and asked ‎them to mention as much ‎pleasant events as possible. ‎When the list reached saturation ‎level, the inappropriate items with ‎respect to legal, cultural and ‎religious concerns were omitted. ‎The final checklist of PCPE ‎consists of two subscales: ‎Frequency (frequency of events ‎during last month) and ‎pleasantness (perceived ‎pleasantness of events). The total ‎score consists of frequency ‎multiplied by pleasantness. To ‎test the reliability and validity of ‎the checklist, the PCPE, ‎Depression, Anxiety and Stress ‎Scale (DASS), the Persian ‎version of WHO Quality of Life ‎and the Demographic ‎Questionnaire were administered ‎in a sample of 104 participants ‎‎(50 male and 54 female).‎

**Results:** Frequency, ‎pleasantness and the total scores ‎of PCPE showed high levels of ‎internal consistency (Cronbach’s ‎alpha, .976, .976 & .974, ‎respectively). Further support for ‎the convergent validity of the ‎PCPE was obtained via ‎moderate negative correlations ‎with depression, anxiety, stress ‎scores in DASS and positive ‎correlation with quality of life as ‎well as respondent’s perceived ‎happiness. There were negative ‎correlations between frequency, ‎pleasantness and total scores ‎and age of the participants ‎‎(Pearson correlation coefficient, r ‎‎= -.194, p<0.05; r = -.270, p<0.01 ‎& r = -.234, p<0.05, respectively).‎

**Conclusion** **‏:‏** **‎** PCPE as an ‎assessment tool has shown to ‎have good reliability and validity ‎among Iranians. Further steps ‎should be taken to validate this ‎instrument in different ‎psychopathologies such as ‎depression, addiction and ‎obesity. ‎

Among psychological interventions for depression, “Behavioral Activation” (BA) has a well-known reputation for its effective role. Several recent meta-analyses (1, 2, 3, 4) have comprehensively documented the efficacy of BA treatments for depression. Pleasant Activity Monitoring and Scheduling (PAMS) is identified as one of the core components of BA along with the assessment of life goals and values, skills training, relaxation training, contingency management, procedures targeting verbal behavior, and procedures targeting avoidance (5). Typically, PAMS serves two functions: Providing information on baseline pleasant activity levels and related moods to inform specific activation assignments, and demonstrating the treatment rationale to the client that there is a meaningful relationship between pleasant activities and mood. In some cases, detailed procedures were also employed to track the relation between activities and mood, including graphs and printouts of the relations that were provided to clients (6).

 Nevertheless, different studies have indicated that PAMS could have positive impacts on reduction of different problematic behaviors including smoking (7), binge eating (8), and ruminative thoughts (9) besides facilitations for cognitive restructuring (10, 11) and significant decrease in depressive symptoms (12, 13, 14).

To implement PAMS, different forms of BA e.g. (15, 16, 17, 6) can be used to train the clients to monitor and schedule their pleasant activities, using Pleasant Events Schedule (PES; 18), a comprehensive list of pleasant activities, or its shortened version either alone or in conjunction with more personal schedules. 

The original English version of PES contains 320 items as a self-report inventory of the frequency of occurrence and subjective pleasantness of typically pleasant or rewarding events over the last month. Although since its development PES has been used in several fields of study (cited by 307 references, for examples please see: 19-22), it is highly culture-bounded and could be considered out of date 30 years after its development. In this study, we aimed to develop and validate a more updated and culturally compatible checklist of pleasant events, which could be used as a pleasant activity monitoring tool in Iranian population.

## Materials and Method

***Ethical issues***:

The study has been conducted after providing sufficient explanation to the participants and obtaining their informed consent. All data are kept confidential and no person will have access to information except

***Participants***:

One hundred twenty-eight participants (female: n = 66) were selected from individuals over 18 years of age who lived in Tehran. The mean age of the participants was 32.3 years (SD = 9.08 years). Twenty-four individuals (female = 12) participated in our three focused group discussions (FGDs) (each with 8 participants), and 104 individuals (female = 54) were asked to complete the Persian Checklist of Pleasant Events developed during FGDs. The participants were recruited using snowball and convenience sampling; all participated voluntarily and without compensation after an oral consent. Also, they were assured of the anonymity of the data.

***Measures:***

Demographic Questionnaire: All Participants were asked to report their demographic data in a researcher-made questionnaire. The required data consisted of age, sex and marital status, number of children, education, job and income. At the end of the demographic questionnaire, the participants were asked two questions: 1. Do you consider yourself as a playful person? (The response options were yes/no); 2. Please rate your happiness on a 0-100 point scale.

Depression Anxiety Stress Scales (DASS): The DASS is a 21-item self-report instrument measuring three main negative emotional states of depression, anxiety, and stress (23). Each of the three subscales consists of seven items; all scored on 0 to 3 scale (range: 0 to 21 for each of the subscales) (23). The Persian version of the DASS has shown to have good psychometric properties in Iranian population (Sahebi, et. al., 2005).

The Persian Version of World Health Organization Quality of Life-BREF (WHOQOL-BREF): To measure quality of life in the sample, WHOQOL-BREF was used. WHOQOL-BREF demonstrated good internal consistency, criterion validity, and discriminant validity (24).

The Persian Checklist of Pleasant Events Schedule (PCPE): In this study, the Persian version of Pleasant Events Schedule was developed and administered. The steps for PCPE development are described in the procedure. 

***Procedure***

**First Phase:**

In the first phase, we held out three focused group discussions (FGDs) to generate the first list of pleasurable items. We defined the concept of pleasant events on the basis of previous literature (Pleasant Events Schedule, PES; 18). According to this definition, pleasant events were those events and/or activities, which produced a sense of pleasure in the individual. In each FGD, participants were asked to mention as much pleasant event and/or activities they could think of. All of the participants’ suggestions were recorded by the researchers on a whiteboard and were then transferred into a word file. During the third FGDs, the generated list, from the data of all three groups, reached the saturation level (i.e., there were no new items to add to the list). Two independent experts were asked to carefully read the items and omit the items which were redundant, ambiguous and inappropriate, not compatible with cultural or religious concerns (for example, getting drunk which is forbidden considering our religious beliefs), and illegal or risky behaviors (for example, taking drugs or driving fast). The remaining 206 items were used in the checklist. Similar to PES, PCPE instructs the individual to rate each item twice, first on a 5-point scale of frequency during the past month, then on a 5-point scale of subjective pleasantness during that month.

**Second Phase** **:**

We used qualitative and quantitative measures to evaluate the content validity of our checklist. To ensure the qualitative validity of our checklist, we asked five independent experts (different from the previous experts) to rate our items on wording, scaling and item-allocation. Their feedback revealed that the items had satisfactory quality. In order to assess the content validity quantitatively, we used Waltz & Bausell’s (25) Content Validity Index (CVI). To assess CVI, we asked 10 experts (different from the previous experts) to rate our items with respect to relevancy, simplicity and clarity on 4-item Likert format. The CVI index for each item was calculated using the number of items which received scores 3 and 4 by the raters for each item divided by the total number of the items (206). According to Waltz and Bausell (25) indexes equal or higher than 0.79 are acceptable. All the items received acceptable scores.

In order to estimate the concurrent validity of PCPE, the correlations between PCPE scores and DASS questionnaire and the Iranian version of WHOQOL-BREF were calculated.

In order to determine the reliability of our checklist, internal consistency and half-split methods were used to calculate PCPE’s reliability. The Cronbach's alpha coefficients were used to calculate internal consistency.

The final checklist of PCPE consisted of 206 items and was administered in a group of 104 participants. Participants were asked to rate the frequency (the frequency of pleasant event occurrence during last month in a five-point Likert format) and the pleasantness (the perceived subjective pleasantness of pleasant events in a five-point Likert format) of each event. We calculated the total score through multiplying the frequency score by pleasantness score for each item. The mean of frequency, pleasantness and total scores for the 206 items for each participant were considered as the final scores for further analysis. The final format of the PCPE plus its English translation as well as self-reported frequency of occurrence and subjective pleasantness of each item is indexed in Appendix 1.

## Results

***Descriptive Results***:

The mean age of the participants was 32.3 years (SD = 8.76 years) with data on age missing for seven participants, and were excluded when calculating the related correlations. Forty-nine participants (47.1%) were married and 50 (48.1%) of them were single, five participants (4.8%) did not answer the marital status question. In response to the question of “Do you consider yourself a playful person?” the answer of 80 (76.9%) participants were “yes”, while 19 participants (18.3%) answered “no” to this question; Five participants (4.8%), did not answer the question; 15 participants (14.4%) had no income, and the income of 59 (56.7%) participants was lower than 10,000,000 Rials and 15 (14.4%) participants had an income of more than 10,000,000; and 15 participants (14.4%) did not answer the question about their income.

As a part of data analysis process, the correlations between frequency, pleasantness subscales and the total score of PCPE with demographic data were calculated using the Pearson correlation coefficient (Table 1). There were no significant relationships between frequency, pleasantness and the total scores and participants’ income. There were statistically significant negative relationships between frequency, pleasantness and the total scores and age of the participants (Pearson correlation coefficient r = -.194, p<.05; r = -.270, p<.01 & r = -.234, p<.05, respectively).

***Reliability Measures*** ***:***

The Cronbach's alpha coefficient for the total scores of the scale was statistically significant (.974). The Cronbach’s alpha for frequency and pleasantness subscales were also statistically significant (.976 and .976, respectively). Half-split coefficients were calculated for the total score of the scale and also for the two subscales. Guttman Split-Half coefficient for the total score of the scale was statistically significant (.905). Guttman Split-Half Coefficients for the frequency subscale was statistically significant (.928). Guttman Split-Half coefficient for the pleasantness subscale was statistically significant (.900).

***Validity Measures:***

There were statistically significant relationships between the two subscales of PCPE (frequency and pleasantness) (Pearson correlation coefficient r =.335, p<.001).

The pleasantness subscale had no significant relationship with any of DASS’s subscales while there were significant relationships between the total score of PCPE and stress (r =.214, P<.05) and quality of life (as assessed by WHOQOL-BREF) (r = .300, p<.01).

There were also significant relationships between PCPE subscales and participants’ responses to the question about their perceived percentage of happiness. The perceived percentage of participants’ happiness had a significant positive correlation with the frequency and total scores of PCPE (r =.360, p<.01 and r =.373, p<.01, respectively). There were also significant positive relationships between participants’ response to the question that whether they considered themselves as a playful person or not and the frequency and total scores of PCPE (r =.318, p<.01 and r =.329, p<.01, respectively).

In order to find the most and least frequently happened events, we sorted the event on the basis of their frequency in our sample. The five most frequently happened events were as follows: Bathing or taking a shower, eating a good meal, wearing clean clothes, thinking about something good in the future and thinking about the person they liked. The five least happened events were as follows: Pottery, carpet weaving, swimming in a river, crocheting, embroidery, or fancy needle work and playing paintball. 

Moreover, we sorted the events on the basis of their pleasantness according to the responses of the participants. The five most pleasant events according to the opinion of the participants were as follows: Completing a difficult task, wearing clean clothes, laughing, eating a good meal, and breathing in a clean air. They also rated these five events as the least pleasant: Crocheting, embroidery, or fancy needle work, weaving, carpet weaving, exchanging Bluetooth files and sewing.

The ranks of each item among all of the items (when sorted in terms of the pleasantness of the item in the study’s sample) and the frequency of each response in the sample (N = 104) are reported in Index 1.

**Table 1:** Correlations of Frequency, Pleasantness, Total scores of PCPE with Demographic and Psychological Data

		**Playful**	**% of** **Happiness**	**Stress**	**Depression**	**Anxiety**	**Quality of Life**	**Age**	**Income**
**Total**	**Correlation**	.329^**^	.373^**^	-.214^*^	-.165	-.013^**^	.300^**^	-.234^**^	-.133^**^
	**Sig. (2tailed)**	.001	.000	.029	.094	.893	.002	.017	.180
**Frequency**	**Correlation**	.318^**^	.360^**^	-.171	-.056^**^	.104	.218^**^	-.194^**^	-.117^**^
	**Sig. (2tailed)**	.001	.000	.083	.570	.294	.026	.048	.236
**Pleasantness**	**Correlation**	.154	.130	-.099	-.131^**^	-.096^**^	.131	-.270	-.046
	**Sig. (2tailed)**	.119	.188	.317	.186	.330	.185	.006	.645

## Discussion

The aim of this study was to develop and validate the psychometric properties of the Persian Checklist of Pleasant Event Schedule (PCPE). As shown in the present study, newly developed PCPE has good psychometric properties. To our best knowledge, PCPE could be considered as the first instrument in its type in Persian. The results with PCPE are largely consistent with those of previous research with the original English version of the PES e.g. (18). For example, the relationship between pleasant events (as reported by PES which is the most similar schedule to our checklist) and mood changes has been established by previous research (26). Grosscup and Lewinsohn (1980) investigated the relationships between the daily occurrence of aversive events, depressed mood, and enjoyment of pleasant events (assessed by PES) in a sample of 21 depressed patients. They found significant relationships between depressed mood and unpleasant events, and between unpleasant events and pleasurableness of pleasant events. Moreover, Wilkinson (1993) found a significant role for pleasant event frequency in daily life in the development of depression. Whisman, Johnson, & Rhee (27) also evaluated genetic and environmental influences on the experience of pleasant events (evaluated by PES (18), depressive symptoms, and their covariation in a sample of 148 twin pairs. Their results indicated that the experience of pleasant events was moderately heritable and that the same genetic factors influence both the experience of pleasant events and depressive symptoms.

The PCPE’s convergent validity was additionally confirmed by the statistically significant relationships of PCPE’s total scores and subscales with respondents’ quality of life and stress. This study also revealed a significant relationship between respondents’ perceived amount of happiness and whether they consider themselves as a playful person and the subscales and the total score of PCPE. The participants’ response to these questions had also negative and significant correlations with depression, anxiety and stress and a positive significant relationship with quality of life which has been supported by previous literature (28). This result is consistent with published studies, which confirmed the relationship between positive events and quality of life e.g. (29).

**Limitations and suggestions**

One of the limitations of this study was that our respondents were selected from the community via sub-optimal sampling methods (snowball and convenience sampling). This limitation lowers the potential for generalization of the present findings.

Finally, one should keep in mind that self-report questionnaires like the PCPE do not assess actual behavior, but rather the personal report of the behavior, which may be influenced heavily by depression or depressed mood itself.

Since there are no published studies using this kind of checklists for Iranians, we suggest that future research on the PCPE include more heterogeneous clinical and nonclinical samples. It is also recommended to use the PCPE in future studies, which are based on the models of behavioral activation. While the length of the schedule was criticized by some respondents in this study, it is also recommended to provide a shorter form of PCPE which may have a more applicability in research areas. While it is an established notion that reminiscence of positive memories can have a positive impact on the person’s mood, it is also recommended to examine the efficacy of PCPE as a mood induction tool. In sum, we conclude that the PCPE can be recommended for further use by clinicians and behavioral activation researchers.

**Figure:**
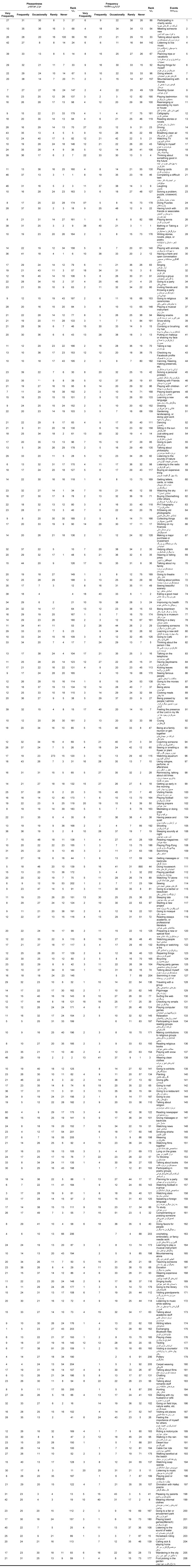


## Conclusion

The findings of this study revealed some significant results, confirming the psychometric properties of our checklist, supporting the notion that this checklist could be used as an instrument in clinical settings to monitor the individual's level of pleasant activities and to suggest several new areas of pleasant activities for behavioral activation purposes. However, compared with the previous literature, the correlations were not satisfactory. These weak correlations could be due to the limitations of the study. Also, since the studies in this area, in which PES has been used as an assessment tool, were mostly done on clinical samples, the reported correlations between indices like stress, anxiety and depression tend to be stronger. The fact that we conducted our study in a non-clinical group may have resulted in lower correlations.

## Index 1

The table below shows the English translation of PCPE’s Items and the original items which are presented in Persian. The table also provides the ranks of each item among all of items (when sorted in terms of the pleasantness of the item in study’s sample) and frequency of each response in the sample (N=104).

**Persian Checklist of Pleasant Events:**

Below you are provided with a list of pleasant events and activities which may occur in everyone’s life. Please read the list and in the first column indicate that how often these events have happened in your life in the past month. In the next column indicate that how pleasant, enjoyable or rewarding was each event during the past month. If an event has not happened to you during the past month, then rate it according to how much fun you think it would have been.

**:سیاهه ایرانی روی دادهای خوشایند**

در زیر فهرستی از رویدادها و فعالیت‌های خوشایند را مشاهده می‌کنید که ممکن است در زندگی هر کسی روی‌دهند. لطفاً فهرست زیر را بخوانید و در ستون اول مشخص نمایید که این فعالیت طی یک ماه گذشته چقدر برای شما اتفاق افتاده است. در ستون دوم مشخص نمایید که این فعالیت‌ها چقدر برای شما خوشایند بوده‌است. ازآن‌جایی که ممکن‌است برخی از این فعالیت‌ها برای شما اتفاق نیفتاده‌باشد، لطفاً تصور کنید که در صورت رخ‌دادن این اتفاق چقدر خوشایند بود.
